# Our Journal for 1887

**Published:** 1886-12-20

**Authors:** 


### EDITORIALS AND MISCELLANEOUS.

### OUR JOURNAL FOR 1887.

*Four Pages of Reading Matter to be Added, and the Day of Publication Changed.*

The present number closes the sixteenth volume of the SOUTHERN MEDICAL RECORD, and though many of our readers seem to have forgotten us in the matter of remitting their subscriptions, we have faith that they will soon attend to this little matter—small to them but important to us who must now meet the many demands of the hard and laborions year now closing. To the journalist the names of his subscribars become so familiar that they seem as intimate personal friends with whom he is accustomed to meet and talk in the daily and social walks of life. We, therefore, extend to each and every one of our readers our fraternal regards as medical men, and the cordial greetings of the season.

And we are gratified to add, that our arrangements for getting out the RECORD the ensuing year are much more satisfactory than heretofore Four pages of reading matter will be added to the journal, and each number will hereafter be promptly issued on the first of the month, instead of the 20th as heretofore· except the next (January) number, which will be necessarily delayed a few days.

Satisfactory arrangements have also been made for original contributions, and great care and attention will be given to the selections and gleanings, so that our readers will be supplied with a brief of all that is useful and practieal in the home and foreign journals. Our aim will be to furnish such an epitome of the medical literature of the World that a careful reader of this one journal will be fairly posted and well up with all the advances in medical science. That we may do this with less labor, and more efficiently, we ask our readers to help us by promptly renewing their subscriptions, paying old arrearages, and by using their influence tc extend the circulation of the RECORD.

